# P-858. Antibiotic Stewardship Needs Assessment in Long-term Care Facilities in Florida

**DOI:** 10.1093/ofid/ofaf695.1066

**Published:** 2026-01-11

**Authors:** Cindy Prins, Venugopalan Veena, Kartikeya Cherabuddi, Cassandra L Johnson, Argentina Charles, A C Burke, Lee Revere

**Affiliations:** University of Central Florida, Gainesville, FL; University of Florida, College of Pharmacy, Gainesville, Florida; University of South Florida - Tampa General Hospital, Tampa, Florida; Nesmith Plastic Surgery, Gainesville, Florida; Florida Department of Health, Tallahassee, Florida; RB Health Partners, Inc, Jacksonville, Florida; University of Florida, GAINESVILLE, Florida

## Abstract

**Background:**

The Centers for Disease Control and Prevention’s Core Elements of Antibiotic Stewardship for Nursing Homes is a guide for antibiotic stewardship (ABS) efforts in long-term care facilities and recommends that the core elements be implemented in a stepwise fashion. To understand the depth of ABS programs in long-term care facilities in Florida, an infection prevention and control needs assessment which included questions about the core elements that have been implemented was conducted.

**Methods:**

The needs assessment was conducted through an online survey from December 6, 2022, through February 9, 2023. Of 302 total respondents, 73 responded to questions about antibiotic stewardship: 80.8% were from skilled nursing facilities/nursing homes (SNF/NH), 15.1% were from assisted living facilities (ALF), and 4% were from continuing care retirement communities or other facilities. Descriptive statistics and bivariate analysis using Fisher’s exact test or chi-square test to compare SNF/NH with ALF were conducted.

**Results:**

SNF/NH were significantly more likely to have an ABS plan than ALF (p< 0.001). While inclusion of core elements varied, those included most by SNF/NH were “Facility has a written statement of support for antibiotic stewardship” (96.6%) and “Facility has an individual who is responsible for stewardship efforts who has training in antibiotic stewardship” (96.6%). Those included least by SNF/NH were “Facility provides education to families on antibiotic use” (73.2%), and “Facility reports antimicrobial stewardship outcome data to staff and providers” (81.0%). The most-used method of tracking antibiotic use was Antibiotic Days of Therapy (75% of responding ALF and 85.5% of responding SNF/NH). Infections preventionists (95.7%) were more likely to rate ABS training as important for themselves than administrators (71.4%) or Assistant/Directors of Nursing (77.8%) were (p=0.055).

**Conclusion:**

All responding SNF/NH in the state had ABS programs that incorporated several of the core elements but few ALF reported having an ABS plan. While facility-specific antibiotic use may not be as high in an ALF as in a SNF/NH, there are over 106,000 ALF beds in the state so implementation of AMS best practices in those facilities may have a positive influence on antibiotic use.

**Disclosures:**

Cindy Prins, PhD, MPH, Becton, Dickinson and Company: Advisor/Consultant Venugopalan Veena, PharmD, Merck: Grant/Research Support

